# Non-destructive Technologies for Plant Health Diagnosis

**DOI:** 10.3389/fpls.2022.884454

**Published:** 2022-05-27

**Authors:** Mervin Chun-Yi Ang, Tedrick Thomas Salim Lew

**Affiliations:** ^1^Disruptive and Sustainable Technologies for Agricultural Precision IRG, Singapore-MIT Alliance for Research and Technology, Singapore, Singapore; ^2^Department of Chemical and Biomolecular Engineering, National University of Singapore, Singapore, Singapore; ^3^Institute of Materials Research and Engineering, Agency for Science, Technology and Research (A*STAR), Singapore, Singapore

**Keywords:** nanosensors, wearable sensors, volatiles, plant health, non-destructive

## Abstract

As global population grows rapidly, global food supply is increasingly under strain. This is exacerbated by climate change and declining soil quality due to years of excessive fertilizer, pesticide and agrichemical usage. Sustainable agricultural practices need to be put in place to minimize destruction to the environment while at the same time, optimize crop growth and productivity. To do so, farmers will need to embrace precision agriculture, using novel sensors and analytical tools to guide their farm management decisions. In recent years, non-destructive or minimally invasive sensors for plant metabolites have emerged as important analytical tools for monitoring of plant signaling pathways and plant response to external conditions that are indicative of overall plant health in real-time. This will allow precise application of fertilizers and synthetic plant growth regulators to maximize growth, as well as timely intervention to minimize yield loss from plant stress. In this mini-review, we highlight *in vivo* electrochemical sensors and optical nanosensors capable of detecting important endogenous metabolites within the plant, together with sensors that detect surface metabolites by probing the plant surface electrophysiology changes and air-borne volatile metabolites. The advantages and limitations of each kind of sensing tool are discussed with respect to their potential for application in high-tech future farms.

## Introduction

Plant health monitoring is an attractive and sustainable strategy that could be used for optimization of crop growth practices. It complements popular agricultural techniques used by farmers to maximize yield including crop rotation, intercropping and genetic modification (Uzogara, 2000; Wang et al., 2014; Yang et al., 2020). It also allows the precise calibration of optimal dosage and application of agrichemicals such as pesticides, herbicides or plant growth regulators (Ang et al., 2021; Roper et al., 2021). However, current chromatography-based analytical techniques are limiting the potential of plant health monitoring in influencing farm management decisions on a day-to-day basis (Pan et al., 2010; Balcke et al., 2012). Though highly sensitive and quantitative, these techniques are destructive and highly labor-intensive, requiring laboratory-based extraction and processing of multiple plant samples for every data point.

The emergence of non-destructive sensors is critical in supporting more efficient plant health monitoring. These sensors transduce plant signals into digital signals to establish direct communication between plants and growers (Qu et al., 2021). By tapping into plants' physiological events in real time, non-destructive sensors enable prompt adjustment of environmental conditions to augment crop productivity while minimizing resource use (Xi et al., 2021). In this mini-review, the focus is on sensors that detect endogenous metabolites, phytohormones and signaling molecules within the plant itself, and sensors that detect surface or air-borne volatile metabolites. Dynamic changes in internal plant metabolites or signaling molecules often influence various aspects of plant growth and development, as well as plant acclimation responses to external stresses. The *in vivo* sensors are based on either electrochemical sensors or plant nanobionic sensors. Both sensing platforms have shown enhancements in sensitivity and selectivity driven by recent advances in nanotechnology which conferred unique electrocatalytic and optical properties to the sensors (Kwak et al., 2017; Li et al., 2021). Table 1 compares the various *in vivo* electrochemical and plant nanobionic sensors, plant metabolites it detects, nanomaterial-based sensor design, detection method and plant species that the sensors were demonstrated in. Besides internal signaling molecules and plant phytohormones, plants also emit surface metabolites in the form of electrical signals, and air-borne metabolites in the form of volatile organic compounds (VOCs) serving as chemical signals that mediate inter-plant communication, and trigger defense responses of neighboring receiver plants (Erb, 2018; Hu et al., 2021). This forms the basis of crop yield enhancement through intercropping. Hence, non-destructive sensors that capture and monitor the emission of VOCs in real-time would also be indicative of plant health, enabling early diagnosis of plant diseases.

**Table 1:** Comparison of the various *in vivo* electrochemical and plant nanobionic sensors.

**Electrochemical sensors**					
**Plant analyte**	**Working electrode**	**Nanomaterials-based modification**	**Detection method**	**Plant species**	**References**
H_2_O_2_	Indium tin oxide	Nano-gold	Voltammetry	Tomato leaves	Sun et al., 2020
SA	Carbon tape	Multi-walled carbon nanotubes/Nafion	Voltammetry	Tomato leaves	Sun et al., 2014
Tryp	Glass carbon	Polydopamine/reduced graphene oxide/MnO_2_ nanocomposite	Voltammetry	Tomato fruits	Gao et al., 2021
Tryp	Miniaturized graphite rod	Multi-walled carbon nanotubes/poly(sulfosalicylic acid)	Voltammetry	Tomato and cherry tomato fruits	Yang et al., 2021
ABA	Ta wires	Vertical graphene with core-shell Au@SnO_2_ nanoparticles assembled onto microneedle array	Chronocoulometry	Cucumber fruits and juices, grapes and radishes, blended Arabidopsis leaf juices	Wang et al., 2021
SA	Al microelectrodes	Core-shell Au@Cu_2_O nanoparticles, graphene and polydopamine densely packed into IDME array	Chronocoulometry	Cucumber leaves, juices and stems	Liu et al., 2021
**Plant nanobionic sensors**					
**Plant analyte**	**SWNT type**	**SWNT modification**	**Detection method**	**Plant species**	**References**
H_2_O_2_	HiPco SWNT and (6,5)-enriched SWNT	Single-stranded DNA oligomer: (GT)_15_	nIR fluorescence quenching	Lettuce, Arugula, Spinach, Strawberry blite, Sorrel, Arabidopsis thaliana leaves	Lew et al., 2020b
NAA	HiPco SWNT	Cationic poly(N-vinyl imidazolium)	nIR fluorescence quenching	Spinach, Arabidopsis thaliana, Pak choi, Rice leaves	Ang et al., 2021
2,4-D	HiPco SWNT	Cationic fluorene-co-phenyl polymer	nIR fluorescence turn-on	Spinach, Arabidopsis thaliana, Pak choi, Rice leaves	Ang et al., 2021
Tannic acid	Monochiral (6,5) SWNT	Polyethylene glycol–phospholipids	nIR fluorescence red-shift and quenching	Soybean Glycine suspension cells, Soybean seedling root exudates, Tococa leaf methanol extracts	Nißler et al., 2022
As (III)	HiPco SWNT	Single-stranded DNA oligomer: (GT)_5_	nIR fluorescence turn-on	Spinach, Rice and Pteris cretica hyperaccumulator fern leaves	Lew et al., 2021
Picric acid	HiPco SWNT and (6,5)-enriched SWNT	Bombolitin II peptide	nIR fluorescence quenching	Spinach leaves	Wong et al., 2017

## Electrochemical Sensors for *in planta* Monitoring of Hormones and Signaling Molecules

Electrochemical sensing technology is a promising strategy for detection of plant hormones and signaling molecules in living plants. The key advantages of electrochemical sensing technologies include good repeatability and accuracy, high sensitivity, portability due to ease of miniaturization, low cost and relatively rapid response times to analytes (Hayat and Marty, 2014). Typically, an electrochemical sensor comprises a sensing or working electrode, a counter electrode and a reference electrode, separated by an electrolyte. In recent years, advances in nanomaterials have resulted in significant enhancement in the analytical performance of these electrochemical biosensors and this has, in turn, opened up more possibilities for rapid and *in situ* detection of analytes in biological samples (Beaver et al., 2021). Carbon-based nanomaterials and metallic nanoparticles are known to enhance biosensor performance and sensitivity due to their unique electrocatalytic properties, facilitating increased electron transfer of redox-active species (Shi et al., 2011, 2012).

**Figure 1:**
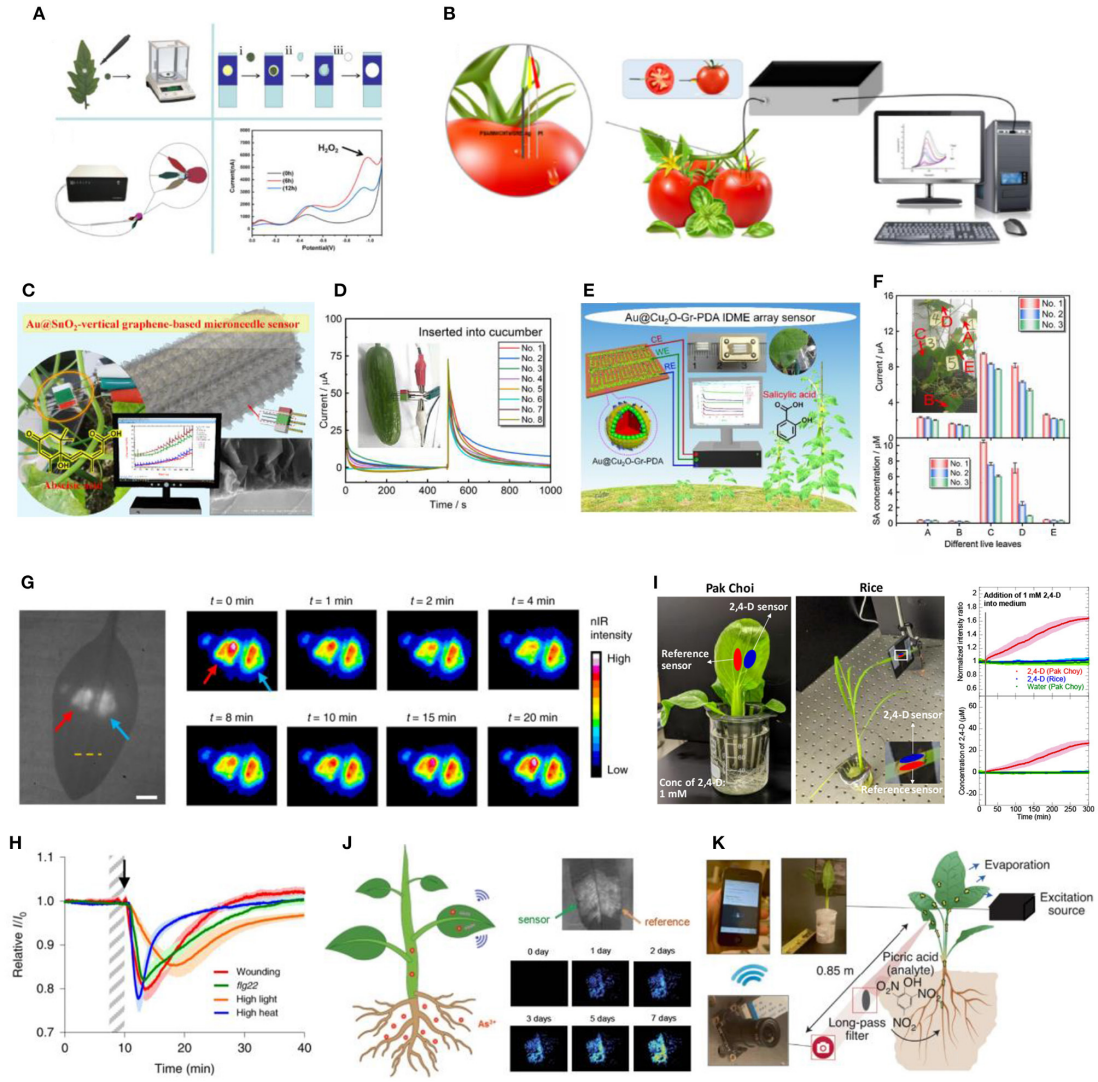
**(A)** Paper-based electro-analytical device used in detection of H_2_O_2_ in circular plant samples punched out of the tomato leaves (Sun et al., 2020); **(B)** Miniaturized electrochemical sensor inserted into tomato fruits for detection of auxin precursor, Tryp (Yang et al., 2021); **(C)** *in situ* ABA electrochemical sensor assembled onto a microneedle array for detection in fruits (Wang et al., 2021); **(D)** Current-time curves generated when the ABA microneedle sensor is inserted into cucumber where ABA concentrations is linearly correlated with the step current observed (Wang et al., 2021); **(E)** *in situ* SA electrochemical sensor arranged in an IDME array for insertion into cucumber leaves (Liu et al., 2021); **(F)** Response current (top) and derived SA concentration (bottom) obtained from the IDME array sensor in 5 different live cucumber leaves (Liu et al., 2021); **(G)** Brightfield (left) and corresponding false-colored images (right) of a spinach leaf infiltrated with H_2_O_2_ (red arrow) and reference (blue arrow) nanosensors on both sides of the leaf mid-vein. False-colored images shows the transient H_2_O_2_ wave upon mechanical wounding of the leaf at t = 0 min (Lew et al., 2020b); **(H)** H_2_O_2_ nanosensor response to different types of stress applied to the plant, including mechanical wounding (red), *flg22* treatment (green), high light (orange) and high heat (blue) stresses (Lew et al., 2020b); **(I)** Real-time sensing of 2,4-D uptake in hydroponically grown pak choi and rice leaves using nanosensors which illustrated a turn-on response observed in pak choi but not in rice over a time-period of 5 h (Ang et al., 2021); **(J)** Arsenite nanobionic sensor infiltrated into hyperaccumulator plant *Pteris creticas* fern, showing intensity changes corresponding to arsenic accumulation detected over 7-day time period upon arsenite exposure (Lew et al., 2021); **(K)** Schematic of standoff detection of nitroaromatic compound, picric acid, using nanosensors with real-time information relayed from the nanosensor-infiltrated plant to a portable Raspberry Pi-based electronic device (Wong et al., 2017).

One such sensing tool is a paper-based electroanalytical device developed for detection of H_2_O_2_ and salicylic acid (SA) in tomato leaves infected with *Botrytis cinerea* pathogen (Sun et al., 2014, 2020). Out of all the reactive oxygen species (ROS) molecules, H_2_O_2_ has the longest stability within plant cells (Huang et al., 2019). Hence, H_2_O_2_ is the key ROS molecule known to participate in cell signaling regulation and induction of plant defense gene expression upon inoculation with bacteria. On the other hand, SA is the main plant hormone involved in plant defense and immunity (Fu and Dong, 2013; Ding and Ding, 2020; Vlot et al., 2021). For detection of H_2_O_2_ or SA on the paper-based electroanalytical devices, circular tomato leaves samples were punched out of the leaf at different times post infection and transferred onto the surface of their respective working electrodes for measurement (Figure 1A). While it provides rapid detection of H_2_O_2_ and SA, this detection method is invasive and destructive as punching out of leaf samples could cause wounding and tissue senescence.

It remains challenging to achieve online monitoring of electrochemical signals *in situ* without the need to extract or cut up leaf samples. Some researchers have managed to insert electrochemical sensors into fruits for measurement of plant metabolites. For instance, an electrochemical tryptophan (Tryp) sensor was fabricated onto a glass carbon electrode (GCE) for detection in tomato fruit samples (Gao et al., 2021). Tryp is an important precursor for auxin (IAA) biosynthesis and IAA is a plant hormone that plays a crucial role in controlling plant development (Teale et al., 2006). Due to the GCE size, the Tryp electrochemical sensor causes plant tissue damage upon electrode insertion in smaller fruits. Recently, a miniaturized Tryp electrochemical sensor has been constructed using a smaller graphite rod electrode (GRE) (Figure 1B) (Yang et al., 2021) which causes less tissue damage during insertion and has successfully detected Tryp levels in smaller fruits such as cherry tomatoes. However, even with the miniaturized GRE, minimizing plant tissue damage when inserting the sensor electrode to other fragile plant parts, such as the leaves or stem, remains complicated.

Microneedle arrays are an attractive option that has been used for construction of minimally invasive electrodes that can be inserted into plant samples. This strategy forms the basis of *in situ* abscisic acid (ABA) (Figure 1C) and SA (Figure 1E) electrochemical sensors (Liu et al., 2021; Wang et al., 2021). ABA is a plant hormone crucial in plant development processes, such as seed germination, stomato closure and plant adaptation to stresses (Lee and Luan, 2012; Hsu et al., 2021). Both sensors use chronocoulometry as electrochemical sensing strategy, which measures the amperometric response currents of the analytes and generates current-time curves. To minimize damage to plant tissues, the ABA and SA sensors were assembled onto a microneedle and inter-digitated microelectrode (IDME) arrays, respectively to be inserted into plant samples such as cucumber fruit (Figure 1D) and leaves (Figure 1F) (Liu et al., 2021; Wang et al., 2021). Remarkably, the SA sensor could be attached to cucumber leaves for 1 month, constantly monitoring changes in SA levels without adversely affect plant growth, confirming its reliability and stability. While attaching the IDME array sensor caused minimal tissue damage to mature cucumber leaves, further work needs to be done to confirm if the same applies to smaller plants or crops.

All in all, electrochemical sensors designed for *in vivo* detection of plant hormones and metabolites are rapid and low-cost. Coupled with novel nanomaterials, the sensors achieved enhanced sensitivities which enable detection of plant hormones and metabolites which are typically present in low quantities. Despite this progress, most electrochemical sensors have been designed to detect biomolecules only in fruit samples with limited applicability to other plant organs. Future approaches include the development of biocompatible nanoelectrodes that could be inserted into the leaves, stem or roots of different plant species with negligible tissue damage.

## Plant Nanobionic Sensors for *in vivo* Monitoring of Hormones and Signaling Molecules

Aside from possessing unique electrocatalytic properties, carbon-based nanomaterials such as single-walled carbon nanotubes (SWNTs) have photostable emission in the near-infrared (nIR) region that does not overlap with chlorophyll autofluorescence (Kwak et al., 2017). This facilitates the application of SWNTs as *in vivo* optical sensors for plant signaling molecules and hormones. The polymer or single-stranded DNA doubles up as a SWNT dispersing agent in aqueous medium and as a synthetic, non-biological antibody for selective recognition and binding to specific plant signaling molecules and hormones. This technique is known as corona phase molecular recognition (CoPhMoRe) whereby different polymer structures or DNA sequences result in the creation of distinct SWNT corona phases that triggers optical modulations such as fluorescence intensity changes or wavelength shifts upon analyte binding (Zhang et al., 2013). Upon syringe infiltration to different plant species, including model species *Arabidopsis thaliana* and non-model plants such as arugula and spinach, these nanosensors could non-destructively monitor the spatiotemporal profile of endogenous signaling molecules and hormones (Lew et al., 2020a). Such information could be captured remotely with portable electronics, providing users with real-time information about plant health. One such nanosensor is designed for *in planta* detection of stress-induced H_2_O_2_ signaling waves in different plant species, including lettuce (*Lactuca sativa*), arugula (*Eruca sativa*), spinach (*Spinacia oleracea*), strawberry blite (*Blitum capitatum*), sorrel (*Rumex acetosa*) and *Arabidopsis thaliana* (Figure 1G) (Lew et al., 2020b). The sensor utilizes a single-stranded (GT)_15_ wrapped SWNT suspension that selectively and reversibly binds to H_2_O_2_. Different types of stress inflicted onto the plants also resulted in the formation of unique H_2_O_2_ signaling waveforms varying in amplitude, velocity and full-width-half-maximum (Figure 1H). The specific stress-induced waveforms aids in the elucidation of complex ROS signaling pathways occurring in real-time upon plant acclimation to external stresses.

Plant nanobionic sensors have also been developed for rapid detection of synthetic auxin plant hormones, used extensively in plant tissue cultures and as herbicides (Figure 1I) (Ang et al., 2021). Synthetic auxins, 1-naphthalene acetic acid (NAA) and 2,4-dichlorophenoxyacetic acid (2,4-D), are important agricultural and horticultural tools as they mimic natural auxins in influencing various aspects of plant growth and development and are more chemically stable and potent compared to natural auxins (Gianfagna, 1995). Separately, 2 different cationic polymer wrapped SWNTs are reported to selectively detect NAA and 2,4-D in different plant species including spinach, *Arabidopsis thaliana, Brassica rapa subsp. chinensis* (pak choi), and *Oryza sativa* (rice) grown in various media, including soil, hydroponic, and plant tissue culture media. The 2,4-D nanosensor also has potential application in rapid testing of 2,4-D herbicide susceptibility as it revealed a discrepancy in uptake and accumulation of supplemented 2,4-D in the leaves of susceptible pak choi vs. resistant rice.

Besides plant hormones and signaling molecules, SWNT-based optical nanosensors have been used in detection of secondary metabolites such as polyphenols. Polyphenols are commonly induced in plants as defense against pathogens or herbivores (Singh et al., 2021). They are prevalent in all plant tissues and organs and are specifically secreted into root exudates to repel pathogenic micro-organisms (Baetz and Martinoia, 2014). Nißler et al. (2022) discovered a selective nanosensor for tannic acid, a key polyphenol using polyethylene glycol phospholipid biopolymer as SWNT wrapping. The optical nanosensor detected tannic acid level changes in *Tococa* leaf methanol extracts challenged with herbivores and in *Glycine max* (soybean) cell culture samples stimulated with a pathogen-derived elicitor, a branched β-glucan cell wall component of the Oomycete fungus *Phytophthora sojae*. It also enabled real-time visualization of polyphenols secreted from the roots of soybean seedlings over a 24 h time-period post elicitor treatment.

By embedding nanosensors into leaves, living plants have also been engineered to detect contaminants that are transported into the plant via the roots and stem. Recently, a plant nanobionic sensor is developed for detection of arsenite, a toxic heavy metal pollutant predominantly found in anaerobic rice paddy soils taken up through silicon transporters in the roots (Ma et al., 2008). Here, the SWNT is wrapped with single-stranded (GT)_5_ which resulted in a strong and selective turn-on response upon detection of arsenite (Figure 1J) (Lew et al., 2021). The sensors were successfully embedded in spinach and rice leaves that detected arsenite that was introduced to the root medium. Further, they were demonstrated in *Pteris cretica* ferns which had the natural ability to hyperaccumulate and tolerate high levels of arsenite (Meharg, 2003). By combining the optical properties of the nanosensor and the intrinsic ability of these ferns to pre-concentrate arsenite, the sensitivity of the nanosensor is enhanced, enabling the detection of 0.6 and 0.2 ppb levels of arsenite after 7 and 14 days, respectively. In another study, peptide-functionalized SWNTs were designed to optically detect picric acid, a common explosive analyte, in spinach plants (Figure 1K) (Wong et al., 2017). In general, the real-time information obtained by these plant nanobionic sensors could be interfaced with portable and inexpensive electronics such as the Raspberry Pi-based camera module, enabling remote sensing in the field.

In summary, plant nanobionic sensors represent a significant advance in the field of non-destructive sensing in living plants. No pre-treatment, extraction or cutting up of plant samples are required as *in vivo* sensing capabilities are imparted to the plants. They are versatile and have successfully extracted spatiotemporal information about various analytes of interest from a diverse range of plant species that are agriculturally important (Lew et al., 2020d). Plant signaling pathways are however complicated and will require the generation of an integrated response from multiplexing of different nanosensors in order to untangle their intricate interactions. In particular, nanoparticle design principles to localize nanosensors within specific plant organs or compartments will be important to facilitate sensor multiplexing and to illuminate inter-organelle signaling (Lew et al., 2018, 2020c).

## Non-Destructive Detection of Surface and Airborne Plant Metabolites

**Figure 2:**
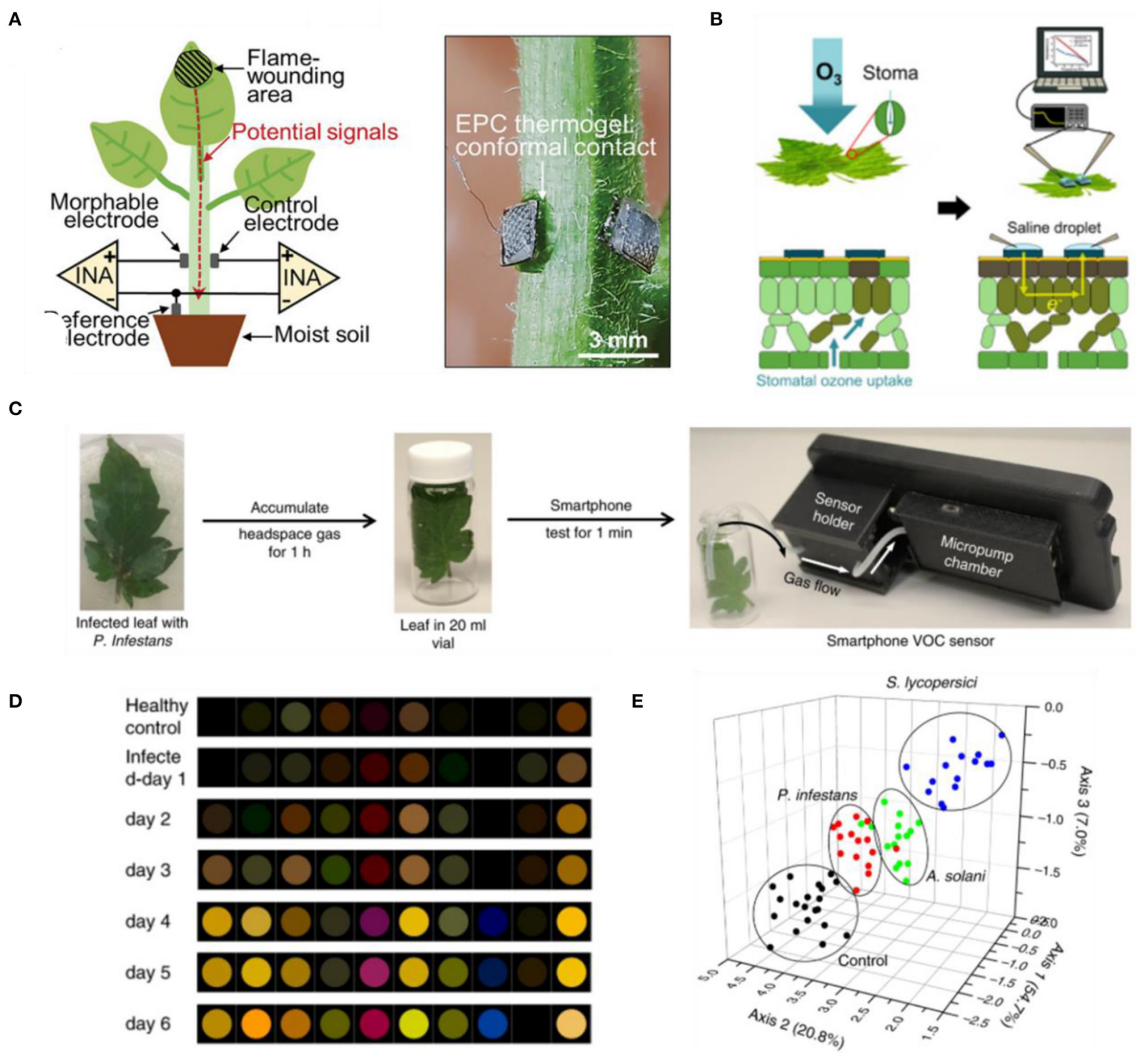
**(A)** Thermogel application to monitor electrical potential signals from plants with hairy stems (Luo et al., 2021). **(B)** Printed conductive polymers enabled impedance spectroscopy to detect ozone damage (Kim et al., 2020). **(C)** Detection of plant VOCs using smartphone-integrated chemical sensor arrays (Li et al., 2019). **(D)** Differential colorimetic response of sensor arrays upon exposure to tomato plants infected with *Pseudomonas infestans* (Li et al., 2019). **(E)** Principal Component Analysis (PCA) plot to distinguish pathogenic infections on tomato plants based on chemical sensor arrays (Li et al., 2019).

Besides internal metabolites, plants also propagate a wide range of signaling molecules along the surface of their organs in response to changing environmental conditions (Mcsteen and Zhao, 2008; Wong et al., 2017; Lew et al., 2020a). These surface metabolites can be accessed non-destructively to inform the state of plant health and stress conditions. In particular, conductive materials which can conform onto the leaf surface have been engineered to probe electrical signals induced by external stresses. These materials have been shown to adhere onto the leaf surface despite the irregular surface topographies and the existence of trichomes in many plant species. Conductive agar gels, connected to metal wires, can be employed as electrodes to capture the temporal profile of electrical signals elicited upon wounding in *Arabidopsis thaliana* (Mousavi et al., 2013; Nguyen et al., 2018). Recently, the conformability of such hydrogel-based approach was improved by using thermogels as morphable electrodes (Luo et al., 2021). The thermogel solution can undergo *in situ* gelation on hairy leaf surfaces at room temperature to provide higher adhesiveness and improved signal-to-noise ratio for plant electrophysiology (Figure 2A). In another approach, biocompatible polymer electrodes were printed on the leaf surface using the vapor-phase polymerization process (Kim et al., 2019). Stress perception would trigger changes in the electrical conductivity along the surface of plant organs, which can be monitored with the vapor-deposited polymer electrodes through bioimpedance spectroscopy. Drought and UV photodamage in plants can be monitored over 130 days with this approach. Through non-destructive impedance measurements, these conformal polymer electrodes also enabled early detection of ozone damage in fruiting plants before the manifestation of leaf necrosis (Figure 2B) (Kim et al., 2020). Instead of monitoring electrical signals propagated by plants, Koman et al. developed an innovative approach to monitor the opening and closing of stomata by printing a conductive ink across the stomatal apertures (Koman et al., 2017). Stomata opening causes the contact of conductive ink across the guard cells to be broken, leading to an increase in the electrical resistance. The circuit is closed when the aperture closes, lowering the resistance. Hence, this approach enabled monitoring of stomata opening and closing latencies. Stomatal dynamics in response to different light wavelengths and drought conditions could be captured with the printed conductive ink over a period of 7 days. These approaches highlight promising technologies capable of long-term monitoring of plant electrophysiology for stress detection. However, they are labor intensive as their measurements necessitate physical contact with conductive wires to obtain the electrical resistance or impedance values. If the circuit information can be transmitted wirelessly, it will pave way toward wider applications in the field without requiring wired connections or skilled personnel to operate such technology.

There are various types of VOCs that plants employ as communication signals in response to abiotic and biotic stresses (Engelberth et al., 2004; Ton et al., 2006; Erb et al., 2015; Acton et al., 2018). As airborne metabolites, these VOCs serve as signaling molecules between different plant organs and between distant plants (Maffei et al., 2011; Karban, 2017; Cofer et al., 2018). Detection of these VOCs could therefore indicate plant health status non-invasively (Tholl et al., 2021). In general, the detection of VOCs from plants in the field is categorized under two sequential procedures: sampling and analysis. Sampling is required to trap and pre-concentrate VOCs to achieve the detection limit of the analytical instrument. An adsorbent material is typically used to capture VOCs, either through static or dynamic pre-concentration (Jansen et al., 2011). Once trapped, these VOCs can be released upon thermal desorption treatment using a gas chromatography coupled with mass spectrometer (GC-MS). The mixture of VOCs can then be separated and identified with GC-MS analysis. However, this conventional GC-MS-based analysis method requires complex laboratory equipment with substantial time lags between sampling and analysis, limiting on-field analysis of plant VOCs. Portable GC-MS instruments have been developed to accelerate VOCs analysis (Beck et al., 2015; Sharma et al., 2019; Stierlin et al., 2020), but they often require manual sample injection and suffer from poor compound resolution due to limited column length.

Electronic nose-based approach has been demonstrated for a more rapid detection of plant VOCs. This technology leverages changes in the electrical output of a chemical sensor array when a mixture of VOCs flow over the sensor array (Cui et al., 2018; Karakaya et al., 2020). The collective array pattern can then be analyzed to distinguish between different VOCs, enabling non-destructive monitoring of plant VOCs. Analysis of VOCs emitted by diseased plants through this electronic nose approach enabled early identification of bacterial diseases in apple plants before symptom manifestation (Cellini et al., 2016), as well as discrimination of healthy rice plants from those infected with brown planthopper (*Nilaparvata lugens*) (Xu et al., 2014). Building on the electronic nose approach, nanoparticle-based chemical sensor arrays were recently coupled with a smartphone for non-destructive analysis of VOCs from tomato plants (Figure 2C) (Li et al., 2019). The arrays would change color differently in response to various VOCs, enabling fingerprinting of 10 green leaf volatiles. This concept was then used to detect late blight in tomato as early as the second day of pathogen inoculation (Figures 2D,E). While these are promising developments in sensing plant VOCs, the stability and selectivity of these technologies in response to different stressors and pathogen infections are still to be studied for widespread application in the field.

## Discussion

A multitude of advanced materials and novel technologies have been proposed for non-destructive plant health monitoring. These toolsets can be broadly categorized into *in vivo* sensors, which aim to probe the signaling molecules within plant tissues, or platforms to detect surface and airborne metabolites. Monitoring the internal signaling molecules has the advantage of detecting physiological concentrations of plant hormones and small molecules immediately after stress is perceived, enabling real-time plant stress detection. Electrochemical-based microneedle array sensors and fluorescent nanosensors are exciting developments in this area which have been employed to study plant signaling pathways and reveal new mechanistic understandings of plant physiology in response to stresses. While promising, these sensors still need to be introduced manually into the plant tissues, limiting the throughput of such approach for agricultural applications. Non-destructive technologies to detect surface and airborne metabolites include conductive polymers and gels to monitor plant electrophysiology, as well as portable GC-MS and electronic nose approach for VOCs analysis. These platforms do not require access to the internal plant tissues or cells, and thus can be conveniently applied outside of the plant organs for non-invasive monitoring. However, some of these approaches suffer from low sensitivity, bulky form factors and unproven demonstrations in the field. Despite these limitations, non-destructive plant health monitoring has significantly improved our understanding of plant physiological responses to external stresses. Progress in this research area should give rise to more advanced technologies which can be applied to study agriculturally relevant crops in the field, bridging the knowledge gap between model plants commonly used in plant biology and economically important crops.

## Author Contributions

MA and TL designed the study, generated ideas for discussion, and wrote the manuscript. All authors contributed to the article and approved the submitted version.

## Conflict of Interest

The authors declare that the research was conducted in the absence of any commercial or financial relationships that could be construed as a potential conflict of interest. The handling editor declared a past collaboration with the authors MA and TL.

## Publisher's Note

All claims expressed in this article are solely those of the authors and do not necessarily represent those of their affiliated organizations, or those of the publisher, the editors and the reviewers. Any product that may be evaluated in this article, or claim that may be made by its manufacturer, is not guaranteed or endorsed by the publisher.
